# Wearable Sweat Loss Measuring Devices: From the Role of Sweat Loss to Advanced Mechanisms and Designs

**DOI:** 10.1002/advs.202103257

**Published:** 2021-10-28

**Authors:** Bowen Zhong, Kai Jiang, Lili Wang, Guozhen Shen

**Affiliations:** ^1^ State Key Laboratory for Superlattices and Microstructures Institution of Semiconductors Chinese Academy of Sciences Beijing 100083 China; ^2^ Center of Materials Science and Optoelectronic Engineering University of Chinese Academy of Sciences Beijing 100029 China; ^3^ Faculty of Hepato‐Pancreato‐Biliary Surgery Chinese PLA General Hospital Institute of Hepatobiliary Surgery of Chinese PLA Key Laboratory of Digital Hepatobiliary Surgery Chinese PLA Beijing 100853 China

**Keywords:** health monitoring, sensible sweat, insensible sweat, wearable sweat devices

## Abstract

Wearable sweat sensors have received significant research interest and have become popular as sweat contains considerable health information about physiological and psychological states. However, measured biomarker concentrations vary with sweat rates, which has a significant effect on the accuracy and reliability of sweat biosensors. Wearable sweat loss measuring devices (SLMDs) have recently been proposed to overcome the limitations of biomarker tracking and reduce inter‐ and intraindividual variability. In addition, they offer substantial potential for monitoring human body homeostasis, because sweat loss plays an indispensable role in thermoregulation and skin hydration. Previous studies have not carried out a comprehensive and systematic review of the principles, importance, and development of wearable SLMDs. This paper reviews wearable SLMDs with a new health perspective from the role of sweat loss to advanced mechanisms and designs. Two types of sweat and their measurement significance for practical applications are highlighted. Then, a comprehensive review of advances in different wearable SLMDs based on hygrometers, absorbent materials, and microfluidics is presented by describing their respective device architectures, present situations, and future directions. Finally, concluding remarks on opportunities for future application fields and challenges for future sweat sensing are presented.

## Graphical abstract

Wearable sweat sensors have received tremendous interest and have flourished due to sweat containing considerable health information. Wearable sweat sensors are summarized with a new health perspective from sweat loss. The potential research directions for the drawbacks of device are then proposed. Finally, concluding remarks on opportunities for unnoticed application fields and challenges for future sweat sensing are presented.

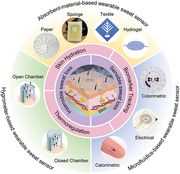

## 1 Introduction

Wearable healthcare devices are rapidly being developed and gradually emerging from laboratory scale to commercial scale for medical applications, which are in the form of miniaturized watches, patches, wristbands, and so on. These wearable devices are designed to provide users with insight into their own physiological state dynamics without heavy equipment and medical professionals in hospitals or laboratories.^[^ ^1^, ^2^, ^3^, ^4^ ^]^ The early application of wearable devices mainly focused on measurement of physical signals (e.g., heart rate, blood pressure, and body temperature). To obtain more in‐depth physiological health information, significant research effort is now being devoted to exploit wearable biochemical devices.^[^ ^5^, ^6^, ^7^, ^8^ ^]^ Such devices can offer individuals a molecular‐level health perspective by noninvasively monitoring biofluids that abound with biomarkers.^[^ ^9^, ^10^, ^11^ ^]^ Among them, sweat shows great promise for wearable real‐time sensing because of its ease of collection and abundant biomarkers.^[^ ^12^, ^13^, ^14^, ^15^ ^]^

The skin is the largest organ of the human body and provides a very large surface for the placement of and interface with a sensor.^[^ ^16^, ^17^ ^]^ Sweat can be divided into two types according to the state of existence and the mechanism of generation in the skin: sensible (liquid) and insensible (vapor) sweat.^[^ ^18^, ^19^, ^20^, ^21^, ^22^ ^]^ Each type of sweat contains a wealth of chemical and dynamic information for monitoring physical and psychological health. For instance, the sensible sweat chloride level test via iontophoresis remains the gold standard for screening cystic fibrosis (CF).^[^ ^23^ ^]^ The concentration of alcohol in insensible sweat has demonstrated a clear correlation with both breath and blood alcohol concentrations, which can be measured to prevent alcohol intoxication.^[^ ^19^, ^24^, ^25^ ^]^ Physiological and psychological stress can be dynamically assessed by analyzing the relation in the concentrations of cortisol, glucose, and vitamin C in sweat.^[^ ^26^ ^]^ In addition, decreased or increased localized sweat loss (rate/volume) is indicative of hyperhidrosis or hypohidrosis, and often assists in stroke diagnosis.^[^ ^27^ ^]^ Therefore, we can track health‐related information from both sensible and insensible sweat using wearable sweat biosensors for sweat extraction, collection, and analysis. Unfortunately, the development of wearable sweat biosensors faces some obstacles about sweat rate, such as complex flow‐rate‐dependent concentration of sweat components, low accuracy of measurement to evaluate known biomarkers, and doubtful reliability of interpretation to explore unknown biomarkers, which limit the application of sweat diagnosis. Therefore, technological developments in advanced wearable forms of sweat loss measuring devices (SLMDs) serve as the foundation for accurate measurement and reliable interpretation of tracking sweat biomarkers and the intrinsic health meaning of sweat loss.

**Figure 1:**
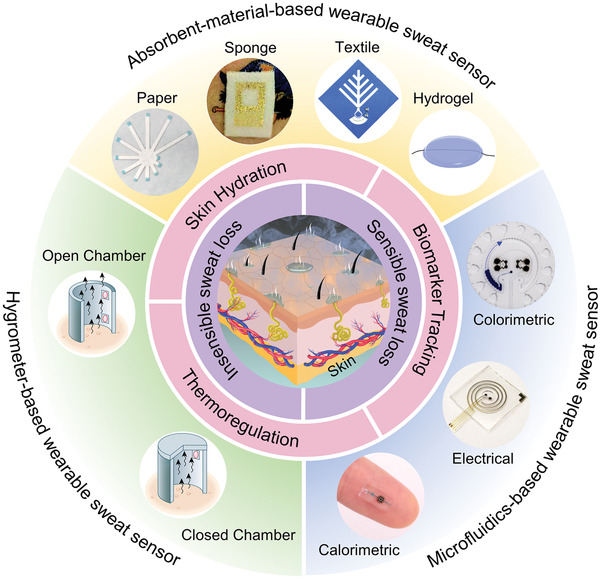
The significance of measuring two types of sweat loss and summary of wearable sweat loss measuring devices based on hygrometer (open chamber method and closed chamber method), absorbent materials (paper: Reproduced with permission.^[^ ^33^ ^]^ Copyright 2019, Springer Nature, sponge: Reproduced with permission.^[^ ^34^ ^]^ Copyright 2014, John Wiley & Sons, textile: Reproduced with permission.^[^ ^35^ ^]^ Copyright 2017, The Royal Society of Chemistry, and hydrogel: Reproduced with permission.^[^ ^36^ ^]^ Copyright 2021, Elsevier), and microfluidics (colorimetric signal: Reproduced with permission.^[^ ^37^ ^]^ Copyright 2019, AAAS, electrical signal: Reproduced with permission.^[^ ^38^ ^]^ Copyright 2018, American Chemical Society, and calorimetric signal: Reproduced with permission.^[^ ^39^ ^]^ Copyright 2021, Springer Nature).

Recently, several good reviews on the progress of sweat capture, collection, and measurement technologies have been reported. However, the framework of many reports usually focused on reviewing sweat collection and analysis for tracking biomarkers rather than sweat loss measurements.^[^ ^28^, ^29^, ^30^, ^31^, ^32^ ^]^ In this article, we present a comprehensive and systematic review of the development of wearable SLMDs for monitoring sweat rate and volume. The paper highlights the importance and development of wearable SLMDs based on different mechanisms and designs. First, we introduce two types of sweat loss from the human body and summarize their secretion volume range and secretion rate range. The performance requirements for designing SLMDs to reliably measure sweat loss based on the sweating range are discussed. In addition, the intrinsic significance of measuring sweat loss in thermoregulation and skin hydration is analyzed, as well as their health significance for accurate measurement and reliable interpretation of sweat biomarker concentrations. In the next section, we systematically divided SLMDs into several categories based on their basic mechanisms, including hygrometer‐based, absorbent‐material‐based, and microfluidics‐based SLMDs (**Figure** **1**). Then, we reviewed some promising SLMDs based on the categories, with emphasis on device designs and performance. Finally, we conclude by highlighting their challenges and opportunities, focusing on research frontiers in sweat sensing.

## 2 Sweat Loss: Sensible and Insensible Sweat

**Figure 2:**
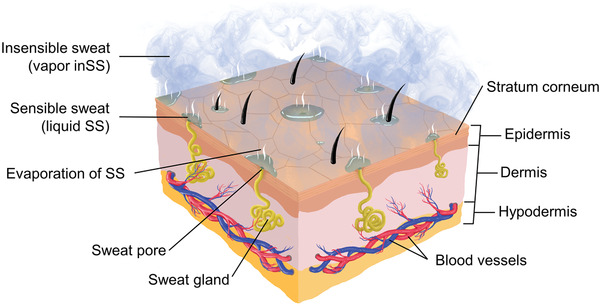
Human skin structure and two types of sweat loss in the skin surface (sensible sweat and insensible sweat).

There are two types of human sweat under normal conditions, as illustrated in **Figure** **2**.^[^ ^21^ ^]^ One is sensible sweat in the form of liquid, which is also known as active sweat or sensible perspiration.^[^ ^40^, ^41^ ^]^ Sensible sweat on the skin surface is mainly produced in most numerous and ubiquitous type of sweat glands, namely eccrine sweat glands, whose activity is controlled by the nervous system in response to physical, thermal, and emotional stimuli.^[^ ^42^, ^43^ ^]^ Many related studies have investigated the mechanism of sweating.^[^ ^31^, ^42^, ^44^, ^45^ ^]^ Recently, sensible sweat has received increasing research attention in the field of wearable devices as it plays an important role in body core thermoregulation and maintenance of skin hydration. By contrast, although the other type of sweat, namely transepidermal water loss or insensible perspiration, has a similar physiological effect in the form of vapor, few studies have used wearable devices to quantify it.^[^ ^46^, ^47^, ^48^ ^]^ In this paper, this vapor type of sweat is referred to as insensible sweat so that its name contrasts with sensible sweat. The entire body of an individual can produce insensible sweat under normal conditions without any external stimulus, and water within the body osmotically diffuses and unconsciously evaporates from the inner dermis and epidermis to the outer stratum corneum (SC) due to the existence of a water gradient.^[^ ^49^ ^]^ Notably, the unstimulated sweat gland can also produce vapor‐type insensible sweat below the threshold condition or under quiescent basal conditions in an unknown fashion.^[^ ^50^ ^]^ Most of the insensible sweat evaporates from the skin surface to the surroundings, whereas a fraction is retained within the SC to maintain skin hydration.^[^ ^51^ ^]^

We summarize the range of human sweat secretion volume and rate reported in medical literature (Section 2.1). When designing SLMDs, the sweating range of the human body sets the performance requirements that should be met for building devices used to measure sweat loss (Section 2.2).

### 2.1 Human Sweat Secretion Volume and Rate

**Figure 3:**
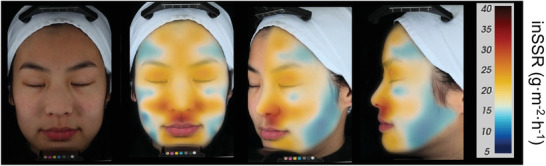
Facial topographic map of inSSR. From left: unmapped subject, anterior view, oblique view, lateral view. Reproduced with permission.^[^ ^57^ ^]^ Copyright 2015, John Wiley & Sons.

Generally, a person's skin surface area is 1.8 m^2^. A person loses ≈0.6–2.3 L of insensible sweat volume (inSSV) daily, which is equivalent to 12–42 g m^−2^ h^−1^. The level of sensible sweat volume (SSV) depends on specific activities, clothing, and ambient temperature. For instance, the SSV is 10–16 L when performing long‐term physical work in a stressful climate.^[^ ^46^ ^]^ Here, sweat is considered to have the same density (1 g·mL^−1^ or 1 mg· μL^−1^) as water because water accounts for 99% of sweat.^[^ ^52^, ^53^, ^54^ ^]^ Additionally, there are significant differences in sweating rates (insensible sweat rate (inSSR) and sensible sweat rate (SSR)) within and between individuals. For inSSR, the whole‐body and regional variability depend on the integrity of the skin barrier function related to the thickness of the stratum corneum, amount of lipid, and so on.^[^ ^55^ ^]^ For instance, aging decreases inSSR in relation to the damage of the stratum corneum, which often causes xerosis cutis in the aged population.^[^ ^56^ ^]^ In regional variability, the hands and feet lose the most (45–90 g m^−2^ h^−1^), the head and neck lose intermediate amounts (20–40 g m^−2^ h^−1^), and all other sites lose lower amounts (10–30 g m^−2^ h^−1^).^[^ ^46^ ^]^ Even in the same body region, inSSR also has apparent variability (**Figure** **3**).^[^ ^57^ ^]^ Remarkably, the unusually direct relationship between thicker stratum corneum and higher inSSR in hand and feet is because the amount of lipid in the stratum corneum, which is important for skin barrier function, is much lower than in other sites.^[^ ^55^, ^58^ ^]^

**Figure 4:**
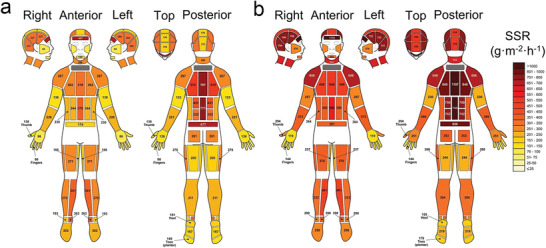
Absolute regional median sweat rates of male athletes at: a) exercise intensity 1 [55% VO_2_ max]; b) exercise intensity 2 [75% VO_2_ max] in moderately warm conditions. Reproduced with permission.^[^ ^60^ ^]^ Copyright 2011, Springer Nature.

For SSR, the secretion rate of each gland makes the difference in SSR in the human body significantly rather than the density of active sweat glands.^[^ ^42^, ^59^ ^]^ Furthermore, measuring sweat rate per gland will offer important knowledge for the relation between component concentration and sweat rate, and thus interpreting inter‐ and intraindividual variability in sweat biomarker concentrations more correctly. Generally, maximal sweat rate per gland is about 4–34 µg gland^−1^ min^−1^.^[^ ^46^, ^59^ ^]^ The density of sweat glands in different positions in the human body is different; for instance, ≈500 glands cm^−2^ in the hand (palm) and foot (sole), 100 glands cm^−2^ in the torso (chest, abdomen, and back), and 60 glands cm^−2^ in the leg and thigh.^[^ ^46^ ^]^ In addition, SSR is also related to its own state. For instance, the intra‐ and inter‐regional range of SSR are ≈90–600 g m^−2^ h^−1^ during passive heating at rest and 200–2000 mg m^−2^ h^−1^ during exercise, which were measured using ventilated sweat capsules.^[^ ^46^ ^]^ A more exhaustive topographic map of SSR is shown in **Figure** **4**.^[^ ^60^ ^]^ In addition to the above factors such as environmental temperature, mood and disease, it also includes humidity, altitude, and solar radiation as external factors, as well as gender, weight, age, and even tattoo as internal factors.^[^ ^61^, ^62^ ^]^

### 2.2 Performance Requirements for Wearable Devices

In general, the total amount of sweat loss is equal to the integral of the sweat loss rate in the measured time. Most studies on SLMDs mainly investigated the different performance requirements of SLMDs by measuring the rate, capacity, and response time owing to the limitations of different mechanisms and designs of wearable SLMDs. In addition to these digital indicators, other nondigital indicators, such as stability, reliability, and user comfort, are also very important for the development of wearable SLMDs.

#### 2.2.1 Sweat Rate

The sweat loss level obtained by measuring sweat rates (SSR and inSSR) is the main characteristic of humidity‐sensor‐based SLMDs, which utilize a humidity sensor to measure the change in relative humidity corresponding to sweat evaporation rate.^[^ ^63^ ^]^ The range of human sweat loss rate is ≈10–2000 g m^−2^ h^−1^ as presented in the previous subsection, where 10–90 and 90–2000 g m^−2^ h^−1^ may represent inSSR and SSR, respectively.^[^ ^46^, ^60^ ^]^ Therefore, a good or ideal SLMD for measuring sweat loss rate should cover this range with good linearity, and has a resolution of at least 10 g m^−2^ h^−1^ to distinguish between inSSR and SSR (the critical sweat rate is ≈90 g m^−2^ h^−1^, which is not fixed and exhibits intra‐ and interindividual variability). Those performance requirements should be adjusted appropriately according to specific application scenarios. For instance, for the skin healthcare of older people, the main measurement parameter is inSSR rather than SSR; therefore, wearable SLMDs with a higher resolution (≈0.1 g m^−2^ h^−1^) are required.^[^ ^64^ ^]^ This requirement is also common in cosmetics and dermatology. Conversely, athletes need wearable SLMDs with a larger measurement range to monitor sweat loss during sports.

#### 2.2.2 Sweat Volume

The sweat loss level obtained by measuring sweat volumes (SSV and inSSV) is the main characteristic of most microfluidics‐based and absorbent‐material‐based SLMDs that utilize the capillarity of microchannels and wicking materials to collect and quantify sweat from the skin surface.^[^ ^65^, ^66^ ^]^ An appropriate volume that will be filled with collected sweat gradually is also required to prevent premature saturation while wearing and using the device. The specific sweat collection volume of the devices needs to be determined and designed according to the inlet size and usage scenario, similar to measurement of the sweat rate.

#### 2.2.3 Response Time

In addition to the requirement for a reasonable measurement range of sweat loss, the intrinsic response time of SLMDs should be smaller than the body's response time to changes in the physiological states. In terms of sweat loss, changes in the physiological states are reflected in changes in the sweat rate and volume. In terms of humidity‐sensor‐based SLMDs, the response of the device can reflect changes in the sweat loss owing to their operating mechanisms, which are based on water vapor pressure and the relatively rapid response time of the commercial humidity sensor. By contrast, other types of SLMDs require an additional experiment to verify their relatively rapid response time. Generally, such an experiment uses a commercial syringe pump to input an adjustable flow rate to mimic sweat injection, and the response of the SLMD is observed to determine whether it can reflect changes in the input flow rate.^[^ ^35^, ^38^, ^67^, ^68^, ^69^ ^]^ This method can also be used to validate SLMDs for precision and accuracy.

## 3 Significance of Measuring Sweat Loss

Sweat performs the functions of thermoregulation and skin hydration, which play an important role in the evaluation of human physiological and psychological status (**Table** **1**). In addition to the intrinsic health significance of these two types of sweat loss, the measurement of biomarker concentration in sweat is also very important (**Table** **2**). Therefore, monitoring and measuring sweat loss is of great significance to improve exercise performance, evaluate skin and emotional health, and assist in disease diagnosis.

**Table 1:** Main intrinsic functions and relevant applications of sweat loss

Functions	Application fields	Specific role	Relevant health instances	Ref.
Thermoregulation	Sports/military/industrial	Elevating athletes/solider/worker performance	Dehydration, hyperhydration	^[^ ^70^, ^71^ ^]^
	Disease	Assisting disease diagnostics/care/treatment	Diabetes, multiple sclerosis, Parkinson's disease, anhidrotic ectodermal dysplasia, secondary hyperhidrosis	^[^ ^42^, ^45^, ^72^, ^73^, ^74^, ^75^ ^]^
	Psychological	Assessing mental health	Primary hyperhidrosis, emotional change, stress	^[^ ^43^, ^76^, ^77^, ^78^ ^]^
Skin hydration	Cosmetic	Testing the efficacy of cosmetics	Topical products, oral supplementation	^[^ ^49^, ^50^, ^79^, ^80^, ^81^ ^]^
	Disease	Assisting disease diagnostics/care/treatment	Atopic dermatitis, psoriasis	^[^ ^21^, ^82^, ^83^ ^]^
	Psychological	Assessing mental health	Emotional change, stress	^[^ ^84^, ^85^ ^]^
		Easing the exacerbation of skin condition	Atopic dermatitis, wound healing	^[^ ^86^, ^87^ ^]^
	Other		Allergic tests, observation of the newborn, skin burns	^[^ ^88^ ^]^

**Table 2:** Typical sweat components: relationship between concentration, SSR, and health role. ×: mixed or uncertain conclusion

Components	Concentration range [mmol L^−1^] in the skin surface	Sweat gland mechanism (main)	Relationship between concentration and rate at higher SSR (major effect)	Physiological role	Health problem correlation	Ref.
Chloride	10–90	Active transport	Direct (reabsorption)	Electrolyte balance	Cystic fibrosis	^[^ ^23^, ^42^ ^]^
Sodium	10–90	Active transport	Direct (reabsorption)	Electrolyte balance	Cystic fibrosis; hypovolemic hyponatremia; muscle cramps	^[^ ^42^, ^94^, ^120^ ^]^
Urea	2–6	Passive transport	Inverse (dilution)	Skin hydration state	Kidney failure	^[^ ^53^, ^102^, ^121^ ^]^
Ammonia	0.5–8	Passive transport	Inverse (dilution)	Acid–base homeostatic	Hepatic disorders	^[^ ^113^, ^122^, ^123^, ^124^ ^]^
Ethanol	0–7	Passive transport	Inverse (dilution)	Body ethanol elimination	Alcohol intoxication; alcoholic cardiomyopathy	^[^ ^19^, ^115^, ^125^ ^]^
Cortisol	0.06 × 10^−5^–0.7 × 10^−5^	Passive transport	Inverse (dilution)	Stress hormone	Physiological stress; psychological stress; psychiatric disorders	^[^ ^115^, ^126^, ^127^, ^128^ ^]^
Lactate	5–40	Gland production	Inverse (dilution)	Skin hydration state	Pressure ischemia; tissue perfusion	^[^ ^42^, ^102^, ^129^ ^]^
Cytokines	0.07 × 10^−9^–1 × 10^−9^ each	Gland production	×	Inflammation marker	Atopic dermatitis	^[^ ^115^, ^130^, ^131^ ^]^
Potassium	2–8	×	×	Electrolyte balance, skin hydration state	Hypokalemia; hypertension; renal failure	^[^ ^42^, ^104^, ^132^, ^133^ ^]^
Calcium	0.2–2	×	×	Sweat secretion, ion resorption	Calcific pancreatitis	^[^ ^115^, ^134^, ^135^, ^136^ ^]^
Bicarbonate	0.5–5	×	Direct (reabsorption)	Acid–base homeostatic	Metabolic acidosis; cystic fibrosis	^[^ ^115^, ^137^, ^138^ ^]^
Glucose	0.01–0.2	×	×	Energy metabolism	Diabetes	^[^ ^115^, ^139^, ^140^, ^141^ ^]^
Uric acid	2–10	×	×	Final metabolite of purines	Gout; cardiovascular and kidney disease	^[^ ^142^, ^143^, ^144^ ^]^
Ascorbic acid	0.001–0.01	×	×	Body metabolism and immunity	Chronic inflammation; scurvy	^[^ ^115^, ^145^ ^]^
Amino acids	≤13 × 10^−3^ each	×	×	Skin hydration state	Lung cancer	^[^ ^45^, ^115^, ^146^ ^]^

### 3.1 Thermoregulation

**Figure 5:**
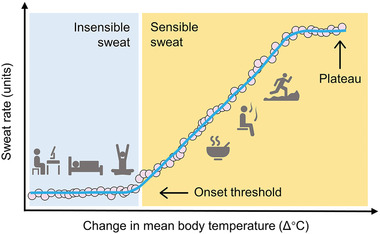
The relationship between change in mean body temperature and sweat rate for thermoregulation. Under the onset threshold (office work, sleeping, yoga, etc.), insensible sweat is the main body sweat loss, where the relationship is characterized by an initially relatively flat portion. Beyond the onset threshold (eating hot food, sauna, running), sensible sweat emerges and becomes dominant, where the relationship is the linear portion. Ultimately, SSR reaches a maximal level, leading to a plateau despite mounting mean body temperature^[^ ^44^ ^]^ (modified from ref. [90]). Reproduced with permission.^[^ ^90^ ^]^ Copyright 2018, John Wiley & Sons.

Thermoregulation is the most important function of sweat loss as it maintains the normal physiological activities of the human body. Under different thermal conditions, the human body with normal thermoregulation always maintains the core temperature within a narrow range of 36–38 °C at rest and up to 41 °C during heavy activities.^[^ ^89^ ^]^ The heat loss caused by the evaporation of the two sweat types plays an indispensable role in thermoregulation. Generally, the control of sweating for thermoregulation is referred to as the integration of core and skin temperatures (mean body temperature).^[^ ^44^ ^]^ Under very mild conditions (e.g., resting, sleeping, and low‐intensity exercise), the dry human body mainly relies on radiation and only needs to evaporate nearly constant insensible sweat to maintain the body temperature. As the thermal load increases and exceeds the onset threshold, the body begins secreting sensible sweat from the sweat gland to the skin surface, which evaporates for heat dissipation, because the latent heat of evaporation of insensible sweat is insufficient to regulate temperature. During this process, the relationship between change in mean body temperature and sweat rate can be briefly described in **Figure** **5**.^[^ ^42^, ^44^, ^90^ ^]^ Thermal challenges that can induce the thermoregulation response of sensible sweat include high‐intensity exercise and sport, passive heat exposure, and intake of hot and spicy food.^[^ ^52^, ^91^ ^]^ Additional details on the physiological mechanism of thermal sweating can be found in other specialized reviews.^[^ ^42^, ^44^, ^89^ ^]^ The secretion rate of sensible sweat increases linearly with an increase in body temperature for heat balance.^[^ ^42^, ^44^ ^]^ In this case, effective cooling of the body is mostly achieved through the latent heat of water vaporization from sensible sweat.^[^ ^92^ ^]^

In terms of the health role of sweat loss in thermoregulation, heat balance at a low core and skin temperature is partly attributed to the evaporation of insensible sweat, whereas heat balance at a high core and skin temperature is mostly attributed to the secretion and evaporation of sensible sweat. Although the secretion and evaporation of sensible sweat play a vital role in proper thermoregulation, excessive sensible sweat loss can increase the risk of dehydration and electrolyte disorder.^[^ ^93^ ^]^ This condition is common in athletes, military personnel, and industrial workers whose sweat loss for thermoregulation exceeds fluid intake during prolonged heat stress.^[^ ^94^ ^]^ In athletes, a low level of dehydration begins to compromise physiological conditions and reduce sports performance. A higher level of dehydration causes heat‐related illnesses and life‐threatening situation.^[^ ^70^ ^]^ Nevertheless, excessive fluid intake, more than required (hyperhydration), can also be fatal.^[^ ^70^ ^]^ Thus, measuring the SSR of athletes and developing fluid replacement strategies would help elevate sports performance while avoiding possible impairment.^[^ ^95^ ^]^

The relative heat balance achieved by thermoregulation can be disrupted if the body or skin is diseased. For instance, diseases with autonomic disturbance that cause a significant reduction in the secretion of sensible sweat, such as diabetes, multiple sclerosis, Parkinson's disease, and anhidrotic ectodermal dysplasia, can severely impair the ability to dissipate heat.^[^ ^45^, ^72^, ^73^, ^75^ ^]^ On the other hand, hyperhidrosis, which produces sensible sweat more than the demand for heat dissipation, can also impair whole‐body thermoregulation.^[^ ^42^, ^96^ ^]^ Hyperhidrosis occurs with primary etiology in the axilla, palms, soles, and face principally involved in emotional sweating in relation to physical and mental stress.^[^ ^43^, ^76^, ^78^ ^]^ It may cause a series of psychological problems and interface the quality of life. Moreover, it also occurs with secondary conditions including pregnancy, drugs, or etiology, including endocrine disorders and malignancies.^[^ ^42^, ^74^ ^]^ Therefore, a significant change in SSR, which is determined using SLMDs, could be used as an effective tool for disease diagnostics and assessing mental health.

### 3.2 Skin Hydration

**Figure 6:**
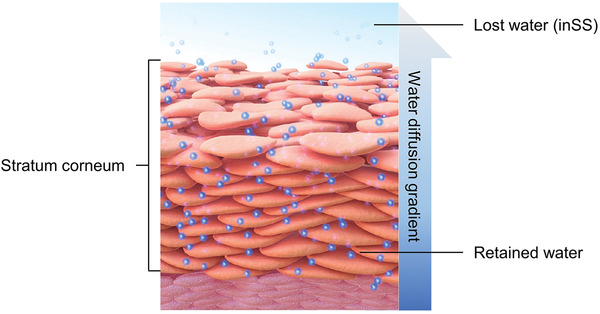
Schematic of stratum corneum and water transport properties for skin hydration (modified from ref. [99]). Reproduced with permission.^[^ ^99^ ^]^ Copyright 2012, Hindawi Publishing Corporation.

An additional function of sweat loss is to maintain skin hydration and skin health. According to the definition of insensible sweat, water is partially retained within the SC owing to its semipermeable structure and the presence of natural moisturizing compounds within corneocytes (e.g., amino acids and their derivatives) to keep the skin hydrated (**Figure** **6**).^[^ ^46^, ^97^, ^98^, ^99^ ^]^ The retained water from insensible sweat is essential for skin health, including skin barrier function, elasticity, and electrical characteristics, and thus influences the overall appearance of the skin.^[^ ^100^ ^]^ Therefore, insensible sweat loss can be a useful way to assess the effects of physical damage, chemical products, and environmental conditions on skin health.^[^ ^49^ ^]^ In general, low insensible sweat loss is indicative of an intact skin barrier function.^[^ ^51^ ^]^

**Figure 7:**
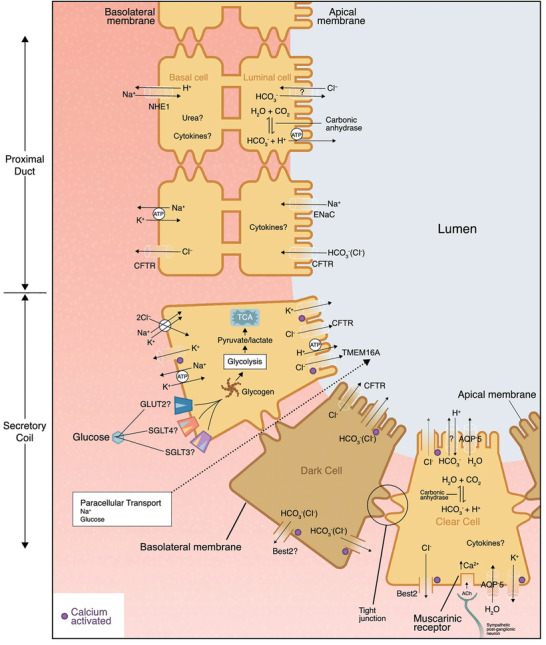
Equivalent schematic of sweat glands and the secretion mechanisms of water and some typical sweat components passage into the secretory coil, including chloride, sodium, potassium, bicarbonate, glucose, etc. Reproduced with permission.^[^ ^115^ ^]^ Copyright 2020, Springer Nature.

The measurement of insensible sweat loss has attracted significant attention in cosmetic, dermatological, and even psychological fields. Cosmetic manufacturers and researchers often use measurements of insensible sweat loss to support the claimed efficacy of their topical products, including product mildness and UV protection.^[^ ^49^, ^50^, ^80^ ^]^ Measurements of insensible sweat loss can also be used to assess the cosmetic benefits of oral supplementation in specific ingredients.^[^ ^79^, ^81^ ^]^ In the dermatological field, elevated insensible sweat loss has been observed due to various skin diseases, such as atopic dermatitis^[^ ^21^ ^]^ and psoriasis^[^ ^82^ ^]^, which can be used in dermatological diagnostics. Furthermore, psychological stress can increase insensible sweat loss and impair skin hydration function and barrier homeostasis, exacerbating existing skin diseases.^[^ ^86^, ^87^ ^]^ Other typical applications include allergic tests, observation of newborns, and supervision of the healing process of skin burns.^[^ ^88^ ^]^

Apart from the importance of insensible sweat loss for maintaining skin hydration, sensible sweat also has great capacity to increase skin hydration, which has often been overlooked.^[^ ^101^, ^102^, ^103^ ^]^ The reason is not only that its water delivery, whose volume is higher than that of insensible sweat, can moisturize the skin more effectively, but it also contains natural moisturizing compounds, such as lactate, urea, sodium, and potassium.^[^ ^104^ ^]^ Monitoring the ability to secrete sensible sweat would be valuable when diagnosing, curing, and treating allergic skin diseases such as atopic dermatitis,^[^ ^83^ ^]^ and when investigating how such diseases progress.^[^ ^103^ ^]^

### 3.3 Biomarker Tracking

The accuracy of measurement of sweat biomarkers is affected by SSR at two different levels. At the biomarker sensing level, the response of sensors is flow‐rate‐dependent owing to mass transport and reaction rates, as well as the activity of the recognition element,^[^ ^12^, ^105^, ^106^, ^107^ ^]^ particularly for electrochemical sweat sensors.^[^ ^108^ ^]^ However, this confounding effect is usually not captured by wearable sweat sensors. Further, at the biomarker generating level, analytes (e.g., urea) that reach the sweat by passive (diffusive) mechanisms of transport could be diluted at a high sweat rate, which can affect the measured results.^[^ ^9^, ^53^, ^109^, ^110^ ^]^ Similarly, dilution effect can also be observed in the compound (e.g., lactate) mainly produced by sweat gland metabolism. Although the higher the sensible sweat rate (the greater the metabolic activity of the sweat gland), the more lactate is secreted, sweat lactate concentration actually decreases with sweat rate due to the dilution effect.^[^ ^42^, ^53^, ^111^ ^]^ Compared with reducing the dilution effect at a lower SSR, active calibration by measuring sensible sweat rates using a detailed perspiration model for the skin and sweat gland is a better approach to correct the dilution effect in more extensive sweat scenarios (e.g., exercise, sport, and sauna).^[^ ^53^, ^112^ ^]^ By contrast, the active portioning components in sweat are essentially not affected by the dilution effect because of the active transport mechanisms.^[^ ^53^, ^110^ ^]^ Nevertheless, the active nature of reabsorption of ions (e.g., sodium, chloride, and bicarbonate) in the sweat gland duct is also confirmed to be flow‐rate‐dependent, such that higher sensible sweat rates are associated with proportionally lower ion reabsorption rates, resulting in higher final concentration.^[^ ^42^, ^113^, ^114^ ^]^ A more detailed secretion mechanisms of sweat components can be referred for **Figure** **7** and other professional reviews.^[^ ^42^, ^115^ ^]^ Therefore, the final concentration of sweat gland components in the skin usually varies with SSR regardless of the sweat gland mechanisms used in producing them, as summarized in Table 2. This indicates that although sensor readouts may be accurate, such biomarkers are heavily dependent on the flow rate at which they are extracted to the skin surface, rather than simply reflecting the biomarker levels in blood and interstitial fluid.^[^ ^12^ ^]^ Hence, if sweat components are to be correlated with biomarker levels in the deeper body, it is necessary to measure and compensate for the effect of SSR.

**Figure 8:**
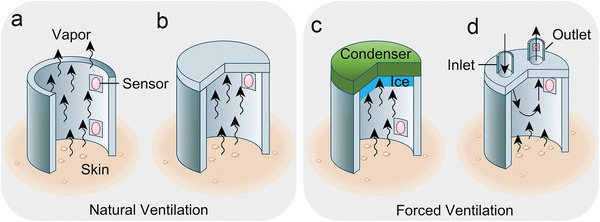
Schematic illustration of operating mechanisms and structures of conventional hygrometer‐based sweat devices. Natural ventilation: a) open chamber method, b) closed chamber method. Forced Ventilation: c) condenser chamber method, d) ventilated chamber method.

The reliability of physiologically meaningful interpretation is essential for exploring and defining sweat components as biomarkers for relevant physiological conditions. To evaluate the possibility of using components as biomarkers related to human health and performance (similar to the sweat sodium and chloride concentrations for CF diagnostics), researchers need to carefully explore the relationship between analyte concentration and sweat loss rate or volume within and among individuals, and normalize concentration with flow rates.^[^ ^67^, ^109^, ^116^ ^]^ Otherwise, the dependence on rates noted in the previous paragraph will increase the statistical variability (the effect of inter‐ and intraindividual differences), and subsequently result in unreliable interpretation of sweat biomarker discovery. Indeed, a current study has shown that accurate determination of the local sensible sweat rate for analyte data normalization can account for the variability within and between individuals.^[^ ^116^ ^]^ This type of normalization is particularly critical for sweat components (Table 2) in which the sweat gland mechanisms have not yet been confirmed (e.g., potassium), which can be employed as potential biomarkers to provide insight into human physiology. In addition, intermittent sweating with periods of activity and inactivity (e.g., heat acclimation) will not only change the SSR, but also relevant component concentrations.^[^ ^117^, ^118^, ^119^ ^]^ Therefore, accurate measurement of SSR can ultimately provide (deterministic) reliable results on how to interpret sweat component concentration data that can be used to reflect physiological health conditions.

## 4 Advances of Wearable Sweat Loss Measuring Devices

Various wearable methods have recently been developed for sweat loss measurement and their operating mechanisms can be simply summarized into three typical categories in accordance with their different ways of acquiring and transducing sweat loss signals. These three types of wearable SLMDs are hygrometer‐based, absorbent‐material‐based, and microfluidics‐based SLMDs. In this section, the basic transduction methods and classification principles of each type of SLMD are introduced to help readers distinguish them effectively. Different SLMDs with sequences from conventional bulky or portable devices to wearable devices are reviewed, focusing on wearable devices with novel device designs to achieve high measurement performances and specific uses, as well as their inherent strengths and weaknesses.

As its name implied, the hygrometer‐based SLMDs mainly rely on miniaturized commercial hygrometers to measure the water vapor pressure (absolute humidity) gradient above the skin surface, which is directly proportional to the vapor flux caused by evaporated sweat.^[^ ^63^, ^147^ ^]^ Then, we can calculate the sweat rate through the relative humidity (RH) rising rate with time from the measured linear part (one hygrometer), or the measured absolute humidity gradient according to Fick's law of diffusion (two or more hygrometers).^[^ ^63^, ^147^, ^148^ ^]^ By contrast, the absorbent‐material‐based and microfluidics‐based SLMDs directly collect and measure sweat loss through the wicking effect of absorbent materials (e.g., paper, sponge, textile, and hydrogel) and the capillarity of microfluidic channel, respectively. They can transduce the sweat loss signal into an electrical signal or a colorimetric signal.

Integrating SLMDs into sweat sensing systems is one of the final goals for practical applications. Compared to the different sensing models (colorimetric or electrochemical models), the specific choice of SLMD design type mainly depends on the substrates of sensing systems (e.g., textiles, papers, and microfluidics). For example, in the sweat sensing microfluidics with different biomarker sensing models, researchers would generally select microfluidics‐based SLMDs for better compatibility.^[^ ^37^, ^38^ ^]^ In the sweat sensing patch based on filter paper, the colorimetric absorbent‐material‐based SLMD is the preferred choice for a simpler structure.^[^ ^149^ ^]^

### 4.1 Hygrometer‐Based Wearable Sweat Devices

Conventional hygrometer‐based SLMDs consist of two parts: a humidity chamber for collecting the sweat‐induced humidity on the skin surface, and one or more hygrometers inside the chamber for detecting sweat rate by measuring either 1) the difference in the absolute humidity of two different chamber positions or 2) the time rise of the relative humidity rise.^[^ ^63^, ^150^, ^151^, ^152^ ^]^ Details about their operating mechanism, development, and suggested conditions for measurement can be found elsewhere.^[^ ^40^, ^63^, ^152^ ^]^ **Figure** **8** and **Table** **3** present a summary of the type and mechanism of conventional hygrometer‐based SLMDs, as well as their strengths and weaknesses. Because the successful commercialization of hygrometer‐based SLMDs provides a paradigm for others, we also introduce several commercial SLMDs and their basic performance based on the operating mechanisms in Table 3.

**Table 3:** Operating mechanisms and typical commercial instruments of conventional hygrometer‐based SLMDs

Method		Operating mechanism			Typical commercial Instrument		
Main	Detailed	Text description	Main strength	Main weakness	Name	Test range [g m^−2^ h^−1^]	Resolution [g m^−2^ h^−1^]
Natural ventilation	Open chamber	It consists of a hollow cylinder chamber with two or more sensors (hygrometer or/and thermometer) inside. The absolute humidity gradient is calculated from sensor readings from different positions, while the vapor sweat is naturally diffused through the open end.	It does not occlude the skin and thus leaves the cutaneous microclimate relatively undisturbed.	It is vulnerable to environmental influences, especially for ambient air flow.	DermaLab (Cortex Technology, Hadsund, Denmark)	0–250	0.01
	Closed chamber	It consists of a chamber with a closed upper end. Sensors in the closed chamber measure the relative humidity rising rate with time caused by collected water vapor from the skin surface.	The closed chamber protects from the disturbance of ambient air flow.	It cannot measure sweat rate continuously because the closed chamber needs to be lifted from the skin to purge the accumulated water vapor regularly.	VapoMeter (Delfin Technology, Kuopio, Finland)	300 (maximum)	0.1
Forced ventilation	Condenser chamber	It consists of a closed chamber at its upper end by a condenser maintained at a temperature below the freezing point of water to remove the water vapor. The humidity gradient can be calculated from two values: 1) the sensor readings mounted in the chamber; 2) the low and stable humidity of the condenser calculated from its temperature.	It maintains a humidity gradient by removing water vapor, enabling continuous measurement of the disturbance of ambient air flow.	Extra condenser control systems with relatively heavy weight and bulky size.	AquaFlux AF200 (Biox Systems Ltd., London, UK)	250 (maximum)	<0.07
	Ventilated chamber/capsules	It consists of a capsule over the skin surface. Influent air/gas of known humidity is forced through the capsule at a fixed flow rate. The humidity and temperature of the effluent air/gas with evaporated water vapor are measured either within or at some point downstream of the ventilated capsule. Then, the humidity difference can be obtained to calculate sweat rate.	It has low analytic variability, high temporal resolution, and wide test range with continuous measurement and without the disturbance of environment.	Extra air pumps and pneumatic systems with relatively heavy weight and bulky size.	Q‐sweat system (WR Medical Electronics Co., Maplewood, MN, USA)	0–200 or 0–1300	<0.02 or <0.08

Researchers have designed a series of wearable hygrometer‐based SLMDs with novel device structures based on the above measurement methods considering emerging wearable and even flexible trends. Given the disadvantages of bulky sizes and increased weight due to additional indispensable systems, forced ventilation methods can hardly be integrated into a wearable format despite their higher accuracy. By contrast, natural ventilation methods are more suitable as wearable prototypes owing to their simple and compact size.^[^ ^151^ ^]^ Therefore, in the early stages of developing wearable hygrometer‐based SLMDs, researchers attempted to design wearable devices directly based on the operating mechanism of natural ventilation methods.

#### 4.1.1 Open Chamber Method

**Figure 9:**
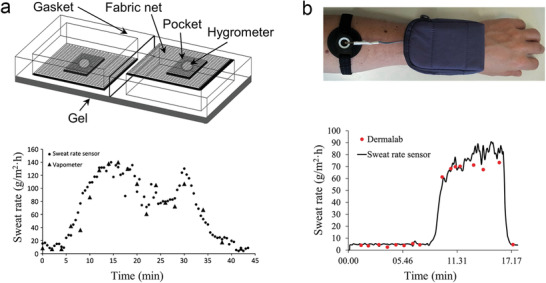
Typical wearable hygrometer‐based SLMDs using the open chamber method. a) Schematic of the first prototype relying on two humidity sensors integrated onto textile substrates and its validation study compared with VapoMeter. Reproduced with permission.^[^ ^153^ ^]^ Copyright 2010, IEEE. b) A wearable SLMD with a rigid open chamber and its validation study compared with DermaLab. Reproduced with permission.^[^ ^95^ ^]^ Copyright 2017, Elsevier.

In 2010, Salvo et al. designed the first prototype of a wearable sweat rate sensor based on the open chamber method and integrated it onto a textile (**Figure** **9**a).^[^ ^153^ ^]^ The difference in the capacitance of the bottom and top commercial humidity sensors has been considered as an indicator of the sweat rate similar to the structure of the open chamber method. However, relying only on humidity sensors to measure the difference in RH cannot yield an accurate gradient of water vapor pressure directly proportional to the sweat rate. Moreover, it is difficult to maintain a fixed distance between two hygrometers owing to their light and flexible structure (a plastic gasket and two fabric nets). For a more reliable measurement, the same research group reported a wearable device with a rigid open chamber to place sensors and did not use the flexible structure (Figure 9b).^[^ ^95^ ^]^ They replaced two hygrometers with sensors that could simultaneously measure humidity and temperature to obtain more accurate results. Their work demonstrated that such wearable SLMDs can be used to monitor the sweat rate of an athlete reliably by comparing it with the measurement obtained using a commercial SLMD.

#### 4.1.2 Closed Chamber Method

**Figure 10:**
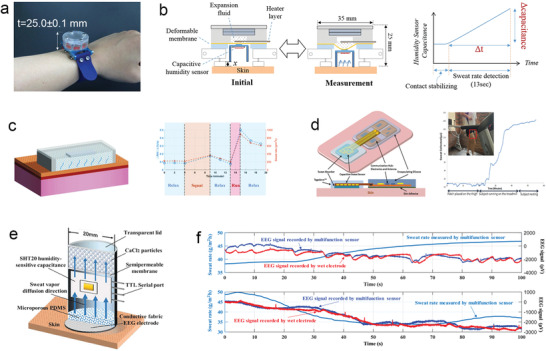
Typical wearable hygrometer‐based SLMDs using the closed chamber method. a) A watch‐type SLMD on human wrist. b) Operating mechanism of the watch‐type SLMD. Reproduced with permission.^[^ ^151^ ^]^ Copyright 2018, Springer Nature. c) Structure and on‐body test of a microporous PDMS sweat rate sensor. Reproduced with permission.^[^ ^157^ ^]^ Copyright 2019, IEEE. d) A NFC‐enabled SLMD based on cellulose sheet. Reproduced with permission.^[^ ^158^ ^]^ Copyright 2013, IEEE. e) Structural diagram of a bifunctional wearable sensor for EEG and sweat rate. f) The simultaneous measurement of EEG signal and sweat rate. Reproduced with permission.^[^ ^160^ ^]^ Copyright 2020, IEEE.

Although the simple structure of the open chamber method is suitable for wearable and continuous measurement, it is vulnerable to environmental effects, particularly for ambient air flows, which affect the humidity gradient in the chamber.^[^ ^154^ ^]^ Thus, an increasing number of researchers have recently developed wearable SLMDs based on closed chamber methods for more practical and diversified scenarios, and designed novel device structures to address the ventilation problem that affects continuous measurement. For instance, Sim et al. proposed a watch‐type sweat rate sensor with low‐weight, low‐power operation, and wind‐resistant characteristics (**Figure** **10**a,b).^[^ ^151^ ^]^ Compared with the device structure of conventional closed chamber methods, a thermopneumatic actuator was integrated above the closed end of the chamber to lift the closed chamber from the skin automatically for ventilation, and hence achieve semicontinuous measurements. The research group developed a series of portable devices for multimodal skin analysis (e.g., skin hardness and conductance) in a single probe based on the novel idea of the automatic actuated chamber.^[^ ^155^, ^156^ ^]^ In the future, such devices for multimodal skin analysis are expected to be wearable, similar to the watch‐type SLMDs they developed.

In addition to the ventilation problem of closed chamber methods, they also have a common intrinsic disadvantage, namely, only the sweat rate in a small range (almost insensible sweat) can be measured because of the contradictory relationship between the measured relative humidity and water evaporation caused by sensible sweat secretion and accumulation. To measure the SSR and eventually extend the test range, Liu and co‐workers used a hydrophilic microporous polydimethylsiloxane (PDMS) in contact with the skin in the developed SLMDs, which were attached to the skin to absorb sensible sweat directly and convert it into air humidity quickly (Figure 10c).^[^ ^157^ ^]^ Similarly, Wei et al. proposed a wearable and conformal system with capability in near‐field communication (NFC) to measure SSR, using cellulose as the sweat absorber (Figure 10d).^[^ ^158^ ^]^ Because the absolute humidity in such closed chambers is related to only the water content in the sweat absorber, the real‐time humidity signal (resistance or capacitance) measured using wearable SLMDs with this structure can reflect the sweat loss level (volume). Although these wearable SLMDs can measure sweat loss in a larger SSR range, they seem to be insensitive to inSSR. The sweat absorbers are disposable and must be replaced after saturation. Moreover, it should be noted that the sweat absorber adjacent to the skin can sometimes lead to occlusion of the sweat ducts and hidromeiosis, which can also occur in absorbent‐material‐based SLMDs.^[^ ^46^, ^62^ ^]^

Inspired by the condenser chamber method, which freezes and removes the water vapor inside, another wearable device structure with similar functions was proposed by Ogai et al.^[^ ^159^ ^]^ It consists of silica gel at the end of the closed chamber as a desiccant, which continuously removes water vapor in the SLMD to prevent accumulation, allowing continuous measurement. In essence, the operating mechanism of this method is similar to the closed chamber method because the sweat rate is still calculated from the change in the readouts of a single hygrometer. Liu and co‐workers designed a hybrid structure for the developed bifunctional wearable sensor using the same method.^[^ ^160^ ^]^ As shown in Figure 10e, this device includes the microporous PDMS mentioned above in the internal sensor to accelerate sweat evaporation, and CaCl_2_ desiccant at the top to remove water vapor and generate a continuous humidity gradient. Furthermore, the forehead electroencephalogram (EEG) signal and sweat rate can be detected simultaneously without mutual interference using an EEG electrode (Figure 10f). Consequently, this bifunctional wearable sensor is expected to provide a more accurate measurement of human physiological conditions. The device design and performance of representative hygrometer‐based wearable SLMDs are summarized in **Table** **4**.

**Table 4:** Device design and performance of wearable hygrometer‐based SLMDs

Method	Device parts and design (from inside to outside)	Test range [g m^−2^ h^−1^]	Resolution [g m^−2^ h^−1^]	Year	Ref.
Open chamber	Gel, fabric net, hygrometer, pocket, gasket	150^a)^ (max)	NA	2010	^[^ ^153^ ^]^
	Adapter, Velcro strips, hard chamber, printed circuit board with the sensors and switch	400 (max)	NA	2018	^[^ ^95^ ^]^
Closed chamber	Skin contact legs layer, humidity chamber, hygrometer, thermopneumatic actuator	3.67–137.68	NA	2018	^[^ ^151^ ^]^
	Microporous PDMS, hygrometer, 3D printed shell	150–1335^a)^	NA	2019	^[^ ^157^ ^]^
	Cellulose, hygrometer, wound dressing	NA	NA	2013	^[^ ^158^ ^]^
	EEG electrode, microporous PDMS, hygrometer, semipermeable membrane, desiccant, 3D printed shell	114 (max)	1.92	2020	^[^ ^160^ ^]^

^a)^ The inferred values base on the chart from their papers.

#### 4.1.3 Existing Situations and Future Direction

From the 1960s to the early 20th century, the development and commercialization of conventional hygrometer‐based SLMDs were the fastest compared with the other types of SLMDs. However, the device structure of hygrometer‐based SLMDs used for vertical humidity measurement is not easy to be thin to construct a flexible system despite their high resolution for measuring sweat rate. Moreover, it can only measure the absolute humidity evaporated by sweat, instead of further collecting secreted sweat for the analysis of sweat components. These shortcomings set bottlenecks to the development of wearable hygrometer‐based SLMDs, which thus lag behind other types of SLMDs in researches in the past ten years. In the future, using miniaturized but still high precise hygrometers is the key research direction to reduce the size of current devices based on a similar operating mechanism with the assistance of micro‐nanomachining technology. Furthermore, it is even possible to implement wearable SLMDs based on the forced ventilated method.

### 4.2 Absorbent‐Material‐Based Wearable Sweat Devices

Advancement in the development of wearable electronic technology has led to the design and manufacture of miniaturized, integrated, and wearable electronic units for signal acquisition, processing, and communication is very mature. In this context, novel absorbent‐material‐based SLMDs developed by combining wearable electronic technology and absorbent patch technique have emerged. These wearable devices offer similar capabilities but without the limitations of conventional methods. Specifically, the sweat loss signal measured using wearable absorbent‐material‐based SLMDs is converted into a readable colorimetric or even continuous electrical signal, not just a preliminary or original weight signal through conventional gravimetric techniques.^[^ ^161^ ^]^ This subsection highlights the current advances in absorbent‐material‐based SLMDs and their novel signal transduction mechanisms in different absorbent materials, including paper, sponge, textile, and hydrogel.

#### 4.2.1 Filter Paper

**Figure 11:**
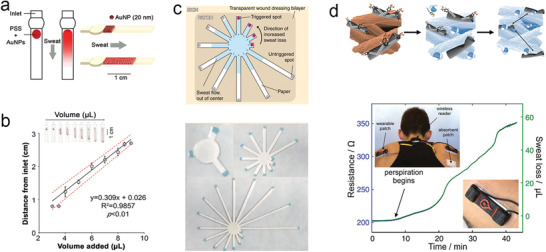
Typical wearable SLMDs by using filter paper materials. a) Design of a sweat volume colorimetric platform. b) Calibration curve of the colorimetric platform for measuring SSV. Reproduced with permission.^[^ ^162^ ^]^ Copyright 2020, American Chemical Society. c) Conceptual illustration and photograph of a finger‐shaped wearable colorimetric patch. Reproduced with permission.^[^ ^33^ ^]^ Copyright 2019, Springer Nature. d) Mechanism and on‐body test of a conductive paper‐based sensor. Reproduced with permission.^[^ ^66^ ^]^ Copyright 2019, John Wiley & Sons.

Filter paper is one of the most common consumables in the laboratory. Compared with other absorbent materials, it has advantages of low cost and good water absorption, and is well suited as a wicking substrate for wearable absorbent‐material‐based SLMDs. Vaquer et al. developed a sweat volume colorimetric platform made of filter paper and simply attached to the skin with tape based on colorimetry.^[^ ^162^ ^]^ As illustrated in **Figure** **11**a,b, dyes are stored in a reservoir, which were carried through the paper strip by the absorbed sensible sweat from the user. Then, the SSV was estimated by additionally gauging the length of the stained paper. The estimated SSV can be used to correct the variation in the colorimetric signal of lactate concentration caused by varying the sweat sample volume. Although its design is simple and easy to scale‐up, the method of data readout is inconvenient and may cause reading errors as a result of the uneven diffusion of the front edge of the dyes.

Jain et al. proposed a wearable patch with an array of markers for easy colorimetric reading and to ensure a convenient method of data readout in real time.^[^ ^33^ ^]^ The patch consisted of laminated filter paper with radially arranged finger‐shaped strips, whose tips had water‐activated dyes as markers. When each strip has been completely saturated with diffused sensitive sweat, the terminal markers are triggered and start changing color, providing an easy readout of SSV for discrete measurements in real time. This novel shape of device design achieved customized fabrication for various systems with different SSV ranges (Figure 11c).

In addition to colorimetry, a wearable resistor based on conductive paper is an ideal structure for continuous measurement of SSV.^[^ ^66^ ^]^ As shown in Figure 11d, the filter paper cellulose can absorb sensible sweat and swell to disconnect the conductive network by coating with single‐walled carbon nanotubes for the conductive network and surfactants to ensure quick water absorption, thereby increasing the resistance. In the future, these paper‐based devices will be available for mass manufacturing owing to their simple and low‐cost designs.

#### 4.2.2 Sponge

**Figure 12:**
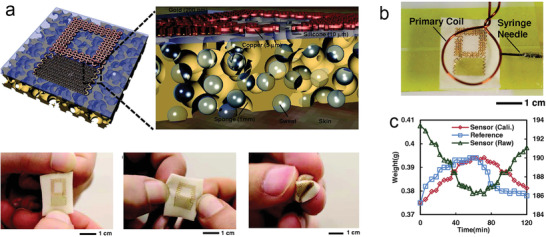
Typical wearable SLMDs by using sponge as absorbent materials. a) A stretchable wireless SLMD based on dielectric detection. b) Image of measurement of dielectric property. c) Body test with comparison to the gravimetric method evaluated using reference absorbent substrates. Reproduced with permission.^[^ ^34^ ^]^ Copyright 2014, John Wiley & Sons.

Another attractive material with low‐cost capability and good water absorption is a sponge. It also has good mechanical properties, including elasticity and stretchability, and is a more suitable alternative as an absorbent material for reducing motion artifacts in wearable SLMDs compared with paper. For instance, Rogers and co‐workers introduced a stretchable and wireless sweat sensor with a novel mechanism for the measurement of SSV (**Figure** **12**a).^[^ ^34^ ^]^ They integrated radio‐frequency identification units on top of the thin sponge in contact with the skin. As the sponge absorbed sensible sweat, the resonance frequency of the SLMD becomes inversely proportional to the water content in the sponge owing to the change in the dielectric property (Figure 12b,c). As a result, such device design enabled wireless quantification of SSVs with good stability in real time. Moreover, other absorbent materials (cellulose paper) have been proposed to construct this SLMD, which indicates that such devices can be designed to meet user's needs. Nevertheless, the sweat absorbed by the sponge can evaporate easily because of the unsealed structure with a porous substrate, which is not suitable for long‐term measurement.

#### 4.2.3 Textile

**Figure 13:**
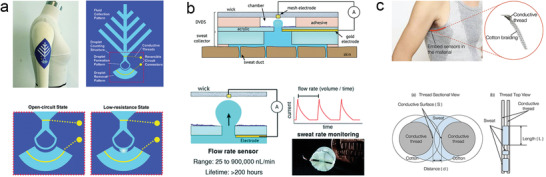
Typical wearable SLMDs by using textile as absorbent materials. Textile: a) a wearable digital flowmeter based on a patterned textile surface. Reproduced with permission.^[^ ^35^ ^]^ Copyright 2017, The Royal Society of Chemistry; b) structure and mechanism of another novel type of digital flowmeter. Reproduced with permission.^[^ ^68^ ^]^ Copyright 2019, The Royal Society of Chemistry; c) a conductive thread‐based textile SLMD integrated into clothing. Reproduced with permission.^[^ ^163^ ^]^ Copyright 2018, MDPI.

Textiles also have an excellent water absorption capability with matured production systems. Unlike the absorbent materials described above, recent SLMDs based on textiles employ a different novel strategy (digital flowmeter) to directly measure SSR instead of SSV.^[^ ^35^, ^68^ ^]^ For instance, Yang et al. proposed the first digital flowmeter based on a patterned textile with contrasting surface wettability (**Figure** **13**a).^[^ ^35^ ^]^ Continuous sensible sweat collected using the textile can be converted into discrete droplets owing to the special pattern design, and then SSR can be obtained by counting the resistance spike caused by the drop of droplets. However, this textile‐based SLMD has a larger size, making it difficult to extend its design to other applications. A more feasible textile‐based SLMD for microfluidic applications was demonstrated by Francis et al. based on a similar operating mechanism.^[^ ^68^ ^]^ As shown in Figure 12b, the sweat droplet entering into the special chamber can increase gradually and short the electrodes, which are then punched off because the droplet is absorbed using the rayon wick. Such a repetitive process is detected as current spikes, which can be calculated as SSR in a period of time. This novel mechanism fills an important research gap in terms of directly determining SSR in absorbent‐material‐based SLMDs.

Apart from expanding textile‐based SLMDs for microfluidics, a thread‐based textile SLMD that can be integrated into clothing without discomfort was developed for continuous SSV measurement.^[^ ^163^ ^]^ The absorbent materials (cotton threads) as well as the conductive thread together and protective layers are weaved for robust SSV measurements. Theoretically, we can determine the SSV by measuring the resistance (voltage) changes between conductive threads similar to the absorbed sweat approach (Figure 13c).

#### 4.2.4 Hydrogel

**Figure 14:**
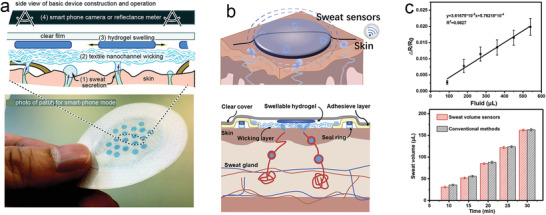
Typical wearable SLMDs by using hydrogel as absorbent materials. a) A wearable SSV monitoring patch and the construction and mechanism. Reproduced with permission.^[^ ^164^ ^]^ Copyright 2020, The Royal Society of Chemistry. b) Design of a wearable strain sensor for measuring SSV. c) Calibration curve of the strain‐enabled SLMD and validation study compared with conventional gravimetric method. Reproduced with permission.^[^ ^36^ ^]^ Copyright 2021, Elsevier.

Hydrogels have set off a new wave of research in the development of SLMDs as they offer a better swelling ratio than other absorbent materials. Various novel hydrogel‐based SLMDs have been designed to measure SSV based on the obvious visible swelling effect of hydrogels. These devices usually use textiles as the substrate to form a hybrid structure to achieve a better performance or specific functions. For instance, Heikenfeld and co‐workers explored a wearable patch consisting of a textile wicking layer, a colored swellable hydrogel for colorimetric detection, and a top film to prevent evaporation and contamination (**Figure** **14**a).^[^ ^164^ ^]^ Textile (rayon) was used to wick and transport sensible sweat from the skin to the upper hexagon hydrogels for better sweat absorption. Meanwhile, white textile can also offer a more contrasting background color for colorimetry than skin tone. The total SSV can be semicontinuously monitored using swollen hydrogels that absorb the transported sweat and measurably increase in geometry. This change in geometry can be detected using two colorimetric modes.

Another typical example is the first prototype of a wearable strain sensor for real‐time SSV monitoring, as reported by Wang et al.^[^ ^36^ ^]^ As illustrated in Figure 14b, this novel device used a conductive fabric embedded in the hydrogel for strain sensing, which could absorb the sweat, swell, and then stretch the fabric to change its resistance. Accordingly, this resistance response is considered an indicator of the absorbed SSV (Figure 14c). Moreover, the textile substrate below the hydrogel not only improves the absorption of this device, but also prevents the embedded strain‐sensing fabric from unwanted stretching caused by natural motions of the skin. The absorbent material and performance of the representative absorbent‐material‐based wearable SLMDs are summarized in **Table** **5**.

**Table 5:** Absorbent materials and performance of wearable absorbent‐material‐based SLMDs. PMMA: poly (methyl methacrylate). NA: no data available. Note: 1) if the content of a column is the same as that of the previous column, we omit it here; 2) in the column of “Test range” and “Resolution,” more comparable values can be obtained from dividing the present values inside by their respective “Inlet areas;” 3) as for wearable absorbent‐material‐based SLMDs and microfluidics‐based SLMD, “material” refers to the type of wicking materials and channel materials, respectively

Category	Material	Signal	Inlet areas	Test range	Resolution	Year	Ref.
				*V*: Volume [µL]; *R*: Rate [µL min^−1^]			
Absorbent‐material‐based SLMDs	Filter paper	Colorimetry	0.04 cm^2^	*V*: 3–9	0.5	2020	^[^ ^162^ ^]^
			0.50 cm^2^	*V*: 5.75–69^a)^	5.75	2019	^[^ ^33^ ^]^
		Resistance	0.28 cm^2^	*V*: 1.5–45.5	NA	2019	^[^ ^66^ ^]^
	Sponge	Resonance frequency	6.16 cm^2^	*V*: NA	36.96	2014	^[^ ^34^ ^]^
	Textile	Resistance spike	18 cm^2^	*R*: 25–245.8	8.3	2017	^[^ ^35^ ^]^
		Current spike	2.27 cm^2^	*R*: 25 × 10^3^–9 × 10^8^	NA	2018	^[^ ^68^ ^]^
		Voltage	NA	*V*: 0–80		2018	^[^ ^163^ ^]^
	Hydrogel, textile	Colorimetry	4.02 cm^2^	*V*: 0–192		2020	^[^ ^164^ ^]^
		Resistance	3 cm^2^	*V*: 90–540		2021	^[^ ^36^ ^]^
Microfluidics‐based SLMDs (colorimetric)	PDMS, PMMA	Colorimetry	7.06 mm^2^	*V*: 50	NA	2016	^[^ ^178^ ^]^
			3.14 mm^2^	*V*: 58		2019	^[^ ^37^ ^]^
			NA	*V*: 79		2019	^[^ ^176^ ^]^
			1.77 mm^2^	*V*: 4.5	0.5^a)^	2019	^[^ ^179^ ^]^
	SIS		0.78 mm^2^	*V*: 60	1.5	2019	^[^ ^183^ ^]^
	PDMS		0.78 mm^2^	*V*: 25	1.3	2019	^[^ ^184^ ^]^
	PU		0.78 mm^2^	*V*: 44.4	NA	2020	^[^ ^186^ ^]^
(Electrical)	PDMS, electrode	Resistance	NA	*V*: 27.2		2018	^[^ ^191^ ^]^
		Admittance	19.62 mm^2^	*V*: 14; *R*: 0.14–2		2018	^[^ ^38^ ^]^
	PET, electrode		19.62 mm^2^	*V*: 24.3		2019	^[^ ^192^ ^]^
	PDMS, PET, electrode		7.06 mm^2^	*V*: 15; *R*: 0.5–3		2019	^[^ ^69^ ^]^
				Customizable		2021	^[^ ^67^ ^]^
	PET, two‐side adhesive tape	Capacitance	6.15 cm^2^	*V*: 31.5		2020	^[^ ^193^ ^]^
	PDMS, Mylar		1.77 mm^2^	*V*: 25.5		2020	^[^ ^194^ ^]^
(Calorimetric)	PDMS	Calorimetry	0.78 mm^2^	*R*: 0–5		2021	^[^ ^39^ ^]^

^a)^ The inferred values base on the chart from their papers.

#### 4.2.5 Existing Situations and Future Direction

Compared with hygrometer‐based SLMDs, the strengths of above wearable absorbent‐material‐based SLMDs for sweat loss measurement can be concluded as threefold: 1) under the premise of low cost, their easy‐access raw materials and relatively simple device structure (especially filter paper) make them easy to scale‐up; 2) the directly measured value is almost SSV which is well suitable for people with excessive sweating behaviors, such as athletes and soldiers; 3) the absorbed or collected sweat can be further analyzed for sweat components, which combines with sweat loss level to afford deeper physiological information. Despite these strengths, their weaknesses are also apparent: 1) existing wearable absorbent‐material‐based SLMDs are relatively difficult to detect insensible sweat in real time, which will restraint their application range; 2) the absorbent material used as the sensing part is disposable; 3) sweat contamination caused by the presence of dead volume between the skin and the devices and background electrolytes of absorbent material itself, which limits the further analysis of sweat.^[^ ^109^, ^165^ ^]^ In terms of the key future direction, researchers should focus on the new absorbent materials with novel structures and biocompatibility, such as the swellable and conductive material with a nanomesh structure.^[^ ^166^, ^167^ ^]^

### 4.3 Microfluidics‐Based Wearable Sweat Sensors

In general, the earliest commercial prototype of wearable microfluidics‐based SLMDs with similar structures can be traced back to a wearable Macroduct (Wescor Inc., Logan, UT, USA), which has a round plastic base with a central hole tightly attached to the skin surface to collect sweat into a coiled plastic tube or channel.^[^ ^168^, ^169^ ^]^ Initially, Macroduct and its larger version, Megaduct (Wescor Inc., Logan, UT, USA), were clinically used with iontophoresis stimulation for sweat collection and confirmatory diagnosis of CF.^[^ ^170^ ^]^ Because the collected sweat in the tube is easily accessible for observation by adding dyes, Macroducts have currently been employed as a useful tool for measuring sweat loss,^[^ ^171^ ^]^ as well as validation study of microfluidics‐based SLMDs. For instance, by labeling the coiled tube with a series of contrasting markers in advance, the SSV filled in the tube can be estimated after image or video recording of the Macroducts.^[^ ^172^, ^173^ ^]^ Furthermore, due to their channel‐type structure and tight contact with the skin, Macroducts also have the scope to prevent sweat evaporation, address swear contamination, and alleviate the hidromeiosis. The above advantages of Macroducts are also observed in recent wearable microfluidics‐based SLMDs owing to their similar microfluidic structure. In the remaining part of this subsection, we will review the representative progress of wearable microfluidics‐based SLMDs in the last five years. In the last five years, the development of wearable microfluidics‐based SLMDs has been the fastest despite their late introduction, and many novel measurement mechanisms have been designed for sweat loss using flexible materials, structures, and circuits. We classify these novel microfluidic devices across three categories according to the type of signal that they transduce sweat loss signals into for a better description, namely, colorimetric, electrical, and calorimetric sweat loss signals. We will also briefly describe their other functions while introducing each microfluidics‐based SLMD. The signal types and performance of the representative microfluidics‐based wearable SLMDs are summarized in Table 5.

#### 4.3.1 Colorimetric

**Figure 15:**
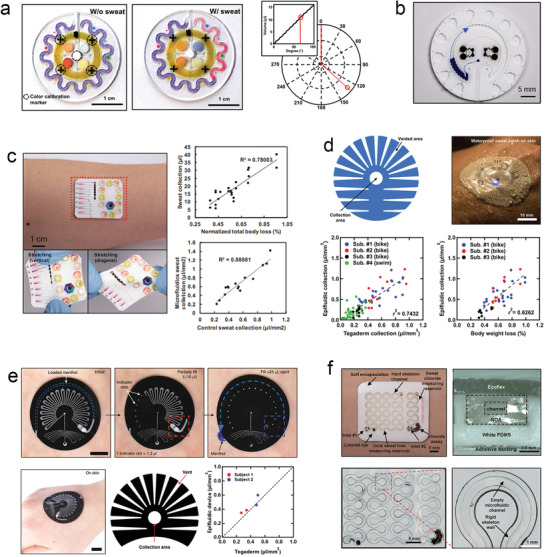
Colorimetric wearable microfluidics‐based SLMDs from Rogers and co‐workers. a) An epidermal system with an orbicular serpentine microchannel for monitoring sweat loss and its operation mechanism. Reproduced with permission.^[^ ^178^ ^]^ Copyright 2016, AAAS. b) Another epidermal system with a circular ratcheted microchannel. Reproduced with permission.^[^ ^37^ ^]^ Copyright 2019, AAAS. c) A soft, flexible skin‐integrated for colorimetric analysis of sweat and its validation study. Reproduced with permission.^[^ ^176^ ^]^ Copyright 2019, American Chemical Society. d) Schematic of a patterned adhesive used in a waterproof, blockage‐reducing sweat patch and its validation study. Reproduced with permission.^[^ ^183^ ^]^ Copyright 2019, AAAS. e) A resettable SLMD with hydration feedback and the operation mechanism and validation study. Reproduced with permission.^[^ ^184^ ^]^ Copyright 2019, Springer Nature. f) Configuration of a wearable SLMD that has a hard/soft hybrid structure for robust measurement of sweat loss. Reproduced with permission.^[^ ^186^ ^]^ Copyright 2020, John Wiley & Sons.

Recently, colorimetric analysis has emerged as the most attractive method for wearable biosensors owing to the relatively straightforward fabrication, simple structure, and easy visual inspection of such device.^[^ ^31^, ^174^, ^175^ ^]^ The colorimetric measurement of sweat loss mainly relies on the water‐soluble dye located near the inlet or filled in channels, which is carried by the collected sweat and passed through the sophisticated microfluidic channel. In this process, a qualitative result might be simply obtained by the naked eye, and a quantitative result would require volumetric labels marked in devices^[^ ^176^ ^]^ or a secondary detection device (e.g., smartphone).^[^ ^177^ ^]^ Moreover, colorimetric measurement of sweat loss does not require rigid electronic units for signal transduction; therefore, the overall device structure can be designed to be softer than other microfluidics‐based SLMDs. As such, many researchers have applied colorimetry to wearable microfluidics to measure sweat loss. Rogers and co‐workers pioneered research in this field and demonstrated a series of typical and forward‐looking works. Therefore, their work is considered to be representative of the latest progress in wearable colorimetric microfluidics‐based SLMDs (their works are summarized in **Figure** **15**, where the data diagram shows the validation study of the corresponding SLMDs).

Rogers and co‐workers usually utilized winding channel designs to increase the total microchannel volume for a given device size to leave more space for additional functions and ensure sufficient volume for sweat loss measurement. For instance, the researchers proposed an orbicular serpentine microchannel filled with water‐responsive dyes to quantify the extent of collected sweat (Figure 15a).^[^ ^178^ ^]^ This serpentine microchannel shape is currently the most common microfluidics‐based SLMD. Similarly, a circular ratcheted microchannel was also developed to determine sensible sweat loss in which the water‐soluble dye was placed near the inlet, as shown in Figure 15b.^[^ ^37^ ^]^ As these dyes begin to change the color of the channel because of incoming sweat, SSV or SSR can be instantaneously determined by image capture according to the channel geometry. Furthermore, additional sensing units, which were surrounded by these circular channel designs, were easily accessible for integration into such devices for multifunctional sweat analysis. The former wearable microfluidic system with a serpentine microchannel encompassed four colorimetric reservoirs for sweat concentration analysis (e.g., pH, lactate, chloride, and glucose) and a sensor for skin temperature.^[^ ^178^ ^]^ In the latter system, lactate and glucose sensors based on biofuel cells were adopted for self‐powered and accurate concentration measurements.^[^ ^37^ ^]^

Further optimization of the microchannel design can result in novel measurement methods for sweat loss with more convenience and functionality. For instance, a relatively long meandering microchannel containing a colorimetric chloride assay was placed between the inlet and in the latter serpentine microchannel (Figure 15c). This novel and multifunctional microchannel can achieve simultaneous measurement of sweat loss and chloride analysis.^[^ ^176^ ^]^ Such an analysis process does not require the use of a reservoir with a fixed volume and is not affected by high SSR. In addition, a variety of volumetric marks were printed near the microchannel based on pre‐estimation, which would help users understand their sweat loss level (SSV) roughly but quickly with the naked eye without a secondary device. Such pre‐estimation of microchannel volume before use has been adopted by an increasing number of microfluidic systems for measuring sweat loss.^[^ ^179^, ^180^, ^181^ ^]^ Of course, more accurate SSV and further estimation of SSR still require image analysis using smartphones.

Although colorimetric microfluidics‐based SLMDs have the advantages described earlier in this subsection, there are also weaknesses associated with their device structure, and Rogers and co‐workers recently addressed some of these weaknesses. One weakness is related to compensatory sweating mechanisms that increase the local SSR due to occlusion of adjacent sweat glands.^[^ ^182^ ^]^ Because the skin adjacent to the inlet is covered by a sealed bottom layer of microfluidic systems, the SSR will increase near the inlet, thereby reducing the accuracy and affecting further analysis. To ameliorate compensatory effects, the researchers introduced a patterned adhesive bonding the SLMD to the skin, as shown in Figure 15d.^[^ ^183^ ^]^ To ensure tight adhesion of the device to the skin, such patterned openings decrease the area of the occluded sweat gland and partly eliminate compensatory effects. Moreover, a unique encapsulation material, poly(styrene–isoprene–styrene) (SIS), which has a lower water permeability and water absorption properties than a conventional PDMS, was utilized in this SLMD to prevent external contamination, interference, and sweat evaporation. Therefore, microfluidics‐based SLMDs with the above designs can measure SSV instantaneously in aquatic settings, thereby extending the application fields of SLMD.

Another weakness is the disposable design for determining sweat loss. After using the colorimetric microfluidics‐based SLMD to monitor sweating activity, the microchannel was stained or its original color was irreversibly changed by dyes, which could no longer be used. This leads to unnecessary waste and decreases user acceptance. To address this, Rogers and co‐workers proposed resettable microfluidics‐based SLMDs, which combined a strain‐actuated elastomeric suction pump to manually expel collected sweat and an elastomeric pinch valve to prevent undesirable sweat ingress into the former (Figure 15e).^[^ ^184^ ^]^ The SSV can be visualized according to pre‐estimated indicator dots using reversible light‐scattering effect (relying on surface microstructure^[^ ^185^ ^]^) without the need for dyes in the serpentine microchannel. Notably, users can manually grasp and pull the bottom of the device to reset and reuse the device below the threshold of the collected SSV. Once this threshold is exceeded, the sweat activates the ejection pump to push a chemical stimulant into the skin, which creates a sensory alert to high levels of sweat loss for users. Moreover, this device also uses a patterned adhesive to eliminate compensatory sweating, similar to the previous SLMD.

In addition, although all the devices mentioned above use soft materials and thin structures capable of good conformality coupling to the skin, external deformations and distortions can easily change the volume of the microchannel. This inherent weakness influences the precision, accuracy, and reliability of the sweat loss measurement. To address this limitation, Rogers and co‐workers formed a serpentine microchannel with a high modulus and low permeability polymers (polyurethane, PU) as the skeleton, as shown in Figure 15f.^[^ ^186^ ^]^ This skeleton structure with a high modulus can efficiently reduce the change in volume of the encompassed microchannel under mechanical loads. Meanwhile, its low permeability is conducive to further sweat analysis in the device. In the future, researchers should focus on designing SLMDs according to application scenarios based on this hybrid structure of the channel to balance the flexibility of devices and measurement stability.

#### 4.3.2 Electrical

Microfluidics‐based SLMDs with electrical sweat loss signals are also a mainstream development direction for measuring sweat loss. Compared with colorimetric microfluidics‐based SLMDs, which provide insight into sweat loss at discrete points in time, electrical SLMDs constitute an attractive operating mechanism with a higher resolution for continuously monitoring sweat loss. In addition, this class of SLMDs does not require careful control and calibration of ambient conditions for accurate readings, such as lighting conditions, which have an inevitable effect on colorimetry.^[^ ^176^, ^177^, ^178^, ^187^ ^]^ Although the above capability is critical for continuous and accurate measurement of sweat loss, there are trade‐offs in power consumption, production complexity, device footprint, and flexibility.^[^ ^188^ ^]^ Generally, the transduction electrical signal used in microfluidics‐based SLMDs includes conductivity (resistance), admittance (impedance), and capacitance. These SLMDs require electrodes embedded into the microchannel to measure electrical signals via an external circuit.

The conductivity or admittance (resistance or impedance) of sweat is associated with the SSR and sweat electrolyte concentrations^[^ ^189^ ^]^ such that sweat and electrolyte loss can be calculated by measuring the conductivity of the collected sweat in the microchannel using electrodes. In 2016, Liu et al. proposed a prototype of a sweat conductivity sensor with a type of wrist watch.^[^ ^190^ ^]^ Two electrode wires were mounted on the vertical channel of the SLMDs to measure sweat conductivity. Its readout can be transmitted via Bluetooth modules and external printed circuits; however, a large size is required, which reduces wearing comfort and hence user acceptance.

**Figure 16:**
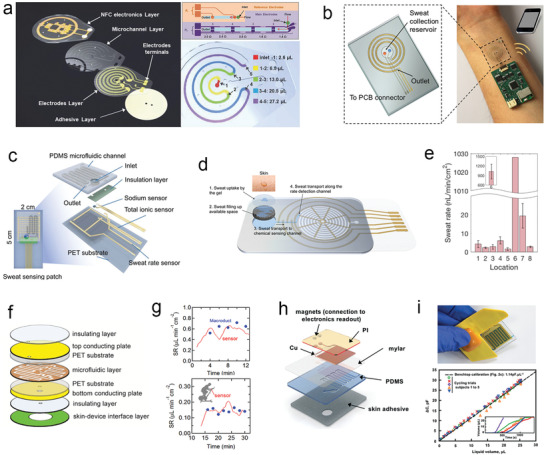
Electrical wearable microfluidics‐based SLMDs. Admittance: a) structure and mechanism of a thin, wireless, battery‐free sweat analysis system. Reproduced with permission.^[^ ^191^ ^]^ Copyright 2018, John Wiley & Sons; b) schematic of a wearable SLMD with a spiral microchannel and a pair of electrodes inside. Reproduced with permission.^[^ ^38^ ^]^ Copyright 2018, American Chemical Society; c) exploded illustration of the various components of a multimodal sweat sensing platform. Reproduced with permission.^[^ ^69^ ^]^ Copyright 2019, The Royal Society of Chemistry; d) structure and mechanism of a wearable patch with capability in continuous collection and measurement of both type of sweat loss; e) sweat loss data measured at 8 different regions. Reproduced with permission.^[^ ^67^ ^]^ Copyright 2021, Springer Nature. Capacitance: f) structural diagram of a low‐cost, wearable capacitive SLMD; g) the validation study and on‐body test compared with Macroduct. Reproduced with permission.^[^ ^193^ ^]^ Copyright 2020, American Chemical Society; h) structural diagram of a wireless wearable SLMD with high sensitivity; i) fully assembled device for measuring sweat rate and its on‐body tests. Reproduced with permission.^[^ ^194^ ^]^ Copyright 2020, The Royal Society of Chemistry.

Recently, flexible printed circuit boards integrated with wireless capabilities have been utilized in wearable devices (as well as electrical microfluidics‐based SLMDs) for low cost and power consumption. A soft, skin‐interfaced microfluidic system was developed by Rogers and co‐workers based on this technology to monitor sweat loss according to sweat conductivity (**Figure** **16**a).^[^ ^191^ ^]^ This system was used to configure a pair of electrodes (main electrodes) with five pairs of probing pads along the serpentine microchannel, and another pair of electrodes (reference electrodes) along a straight microchannel. These two electrodes can collect sweat and measure sweat resistance. Specifically, the pairs of pads with different locations define the total measured SSV, and the SSR and ion concentration can be calculated from the impedances of the two electrodes. The researchers further developed a microfluidic system with additional integrated quantitative immunoassays based on a similar measuring mechanism for cortisol and fluorescence assays for glucose and vitamin C.^[^ ^26^ ^]^ This system, combined with the measurement of sweat loss and skin conductivity, has the potential to define human dynamic stress conditions with good sensitivity. Although the above method can reliably measure sweat loss and ion concentration, the method of determining the results is tedious, and the device structure with an external readout circuit is complex.

A simplified and excellent method is to directly quantify the SSV based on the admittance between the electrodes related to the distance traveled by sweat. Specifically, as sweat travels along the microchannel, the admittance magnitude increases with increasing SSV owing to a decrease in the effective resistance and an increase in the capacitance.^[^ ^38^ ^]^ In addition, because admittance is associated with ion concentration in sweat, it needs to be determined to calibrate the measured SSV. Javey and co‐workers configured a pair of electrodes along a spiral microchannel in their developed microfluidics‐based SLMDs using this approach (Figure 16b).^[^ ^38^ ^]^ A sodium electrochemical sensor was located at the opening of the device for sweat sodium concentration analysis and SSV or SSR calibration. Similar to the above device design, the researchers recently demonstrated this type of device for high‐throughput and cost‐effective fabrication via roll‐to‐roll processes.^[^ ^192^ ^]^

In addition to configuring the entire microchannel with electrodes, the electrodes for the admittance signal can also partly and regularly make contact with the microchannel to measure sweat loss without interference from varying ion concentrations.^[^ ^67^, ^69^ ^]^ Javey and co‐workers designed an admittance SSR sensor consisting of two interdigitated finger electrodes through which a serpentine channel passes partly and repeatedly (Figure 16c).^[^ ^69^ ^]^ The collected sweat accumulating in such structures passed and connected the electrode fingers gradually, and the admittance measured between the electrodes discretely jumped. By counting the jumps of the admittance signal in a time interval, SSR can be calculated according to known distances between each finger, which can also discretely quantify the SSV simultaneously. Although sweat ion concentrations affect the admittance magnitude of the signal jumps, they do not affect the periodicity (time intervals) of the jumps, which is only determined using the electrode structure.

The researchers also developed a small footprint sweat rate sensor with two interdigitated wheel shape electrodes aligned with a spiral microchannel based on this innovative method.^[^ ^67^ ^]^ As shown in Figure 16d, the sweat can be rapidly absorbed at low secretion rates (as low as 5 g m^−2^ h^−1^) by combining a hydrogel with a hydrophilic filler in the sweat collection area. Moreover, the tailored microchannel with sufficiently small dimensions (cross‐section) provided sufficient pressure to push this low rate of sweat into the device, and relatively fast movement of collected sweat within the channel. Such special microchannels with upper radial electrodes are crucial not only for capturing low sweat volumes, but also for selective and continuous sweat rate measurements. According to the measurement data provided in this work, continuous collection and measurement of both SSV and inSSV at rest are realized (Figure 16e), which satisfies the need to measure both types of sweat loss simultaneously in the field of microfluidics‐based SLMDs. Notably, the device parameters can be altered to improve measurement performance. For instance, the primitive eight radial electrodes in their SLMD can be increased to 24 radial electrodes for higher accuracy and enhanced time resolution. By incorporating the detection of specific sweat biomarkers, this microfluidic system proved to be an ideal tool for monitoring special user groups (elderly and office workers) during daily routines.

In addition, Choi et al. proposed a low‐cost and easy‐to‐fabricate capacitive SLMD that is somewhat similar to a parallel‐plate capacitor.^[^ ^193^ ^]^ Its simple device consists of two conducting parallel plates on polyethylene terephthalate (PET), plastic insulating layers, and a central microfluidic layer with a channel (Figure 16f). During measurement, the original air‐filled area between the two parallel electrodes is increasingly replaced by water as sweat travels along the channel. As a result, it increases the capacitance between the two electrodes owing to significant difference in the relative permittivity of air (*ε*_r_ of air = 1) and sweat (*ε*_r_ of sweat ≈ 80). While this device had a well‐established linear relationship between the change in capacitance and the measured SSV, some on‐body test results differ from those measured using Macroducts (Figure 16g), which indicates that the accuracy of the SLMD is not good. Similarly, Rogers and co‐workers proposed a more reliable capacitive method with an elaborate structure. The sensitivity was optimized by inserting an ultrathin layer (Mylar) between the microchannel and soft interdigitated electrodes as a sensing interface, which produced sufficiently large changes in capacitance for accurate measurement of sweat loss (Figure 16h,i).^[^ ^194^ ^]^ As the microchannel was filled with sweat, the area of the water‐filled zone below the electrodes increased, resulting in changes in capacitance relevant to SSV measurement.

#### 4.3.3 Calorimetric

**Figure 17:**
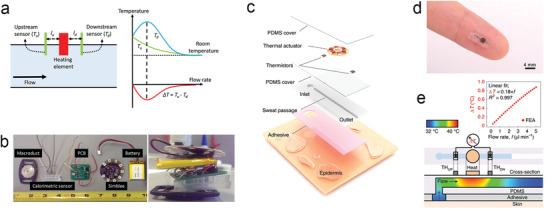
Calorimetric wearable microfluidics‐based SLMDs. a) Theoretical principle of the calorimetric flow rate sensor. Reproduced with permission.^[^ ^197^ ^]^ Copyright 2017, AIP Publishing. b) Separate parts and overall device of a wireless SLMD for calorimetric measurement of sensible sweat loss. Reproduced with permission.^[^ ^71^ ^]^ Copyright 2018, MDPI. c) Structural illustration of a miniaturized, on‐skin, flexible SLMD for wireless sensing of sweat loss. d) Image of the thermal flow rate sensor on a finger. e) Operating mechanism of the calorimetric SLMD for measuring sweat rate. Reproduced with permission.^[^ ^39^ ^]^ Copyright 2021, Springer Nature.

Calorimetric sensors have been extensively used as good tools for determining the flow rate, particularly microflow sensing,^[^ ^195^, ^196^, ^197^ ^]^ with the potential for measuring the sweat rate. In this type of device, a number of thermistors were placed at different locations (upstream or downstream) around a heater heated to a constant temperature to measure the temperature. Consider the simplest and most commonly used two thermistor structure as an example (**Figure** **17**a). If there is no fluid flow, the temperatures of the symmetrical upstream and downstream thermistors will be the same. As the fluid flows, the temperature in the upstream thermistor begins to decrease owing to the cooling effect of the fluid flow, whereas the temperature in the downstream thermistor begins to increase because the heater temperature gradient is forced toward (adverted) downstream by the flow. In this process, the temperature difference (Δ*T*, downstream minus upstream) is a monotonously increasing function of the flow rate. However, as the flow rate exceeds a critical maximum, Δ*T* decreases because the cooling effect of the fluid flow overcomes the heat, which starts to cool down.^[^ ^197^ ^]^ Therefore, the device design needs to be optimized to ensure that Δ*T* corresponds to only one flow rate within the desired measurement range.

Kaya and co‐workers proposed a wireless SLMD with calorimetric readout signals for measuring SSR based on this operating mechanism.^[^ ^71^ ^]^ This novel system consisted of a Macroduct for collecting sweat and filling subsequent calorimetric flow rate sensor^[^ ^197^ ^]^ and a wireless platform for tracking SSR in real time (Figure 17b). However, it has a high level of power consumption and a large overall system. Recently, Rogers and co‐workers have made an important progress in calorimetric wearable SLMDs.^[^ ^39^ ^]^ A wireless, reusable, and miniaturized flow rate sensor (smaller than a fingertip) was developed based on a similar operating mechanism of calorimetry, as illustrated in Figure 17c–e. It consisted of a power‐efficient thermal actuator and two symmetrical precision thermistors, which were all thermally coupled to the sweat but are not directly in contact, for measuring the sweat flow rate. Below these thermal components, a simple, short, and straight microchannel with inlet and outlet was used to collect and measure sweat loss without the limits of volume capacity, similar to the above colorimetric and electrical SLMDs. Meanwhile, this calorimetric wearable microfluidics‐based SLMD, combined with an external circuit for processing and wireless transmission of sweat loss signal, achieved automatic and continuous measurement of SSR and SSV, and was validated using its existing colorimetric SLMD with matured technology.

In terms of the future research direction of calorimetric wearable SLMDs, we believe that this method can be developed to become an ideal microfluidics‐based SLMD capable of simultaneous, instantaneous, and reusable measurements of both SSR and inSSR through the optimization of current device design. Specifically, the structure presented by Javey and co‐workers can be utilized at the inlet and microchannel.^[^ ^67^ ^]^ At the inlet, both types of sweat loss can be absorbed using a patterned filler coated with a saturated hydrogel. At the microchannel, the absorbed sweat can be pushed by the capillary action into a small cross‐section channel, which is also crucial to ensure a fast flow rate to generate a sufficient temperature difference for detecting when the sweat rate is low (inSSR).

#### 4.3.4 Existing Situations and Future Direction

Compared with other types of wearable SLMDs, the unique advantage of microfluidics‐based SLMDs is that they can collect the disordered sensible sweat and even the diffusive insensible sweat on the epidermis via capillary action of the microchannel, thereby generating an orderly and controllable fluid flow. The created flow rate is directly proportional to the initial sweat rate. Subsequently, it can be detected by a flow rate sensor mounted in the microchannel for determining SSR and inSSR as well as their relevant sweat volume. Nonetheless, using this unique advantage for developing microfluidics‐based SLMDs has not yet received much attention to date. Toward sweat rate sensing in the microchannel, current micromachined flow sensors were considered as a powerful candidate with the support of advanced processing and transferred technology.^[^ ^198^, ^199^, ^200^ ^]^

## 5 Conclusion

In conclusion, this paper presented a review of progress in wearable devices for measuring sweat loss from a new health perspective of sensible and insensible sweat. We classified the different operating mechanisms and device designs, namely hygrometer‐based, absorbent‐material‐based, and microfluidics‐based wearable SLMDs. These wearable devices, particularly microfluidics‐based SLMDs, have attracted increasing attention and made significant progress owing to the important health significance of sweat loss. Yet most of the existing wearable SLMDs only offer good application prospects for some specific scenarios owing to their inherent shortcomings such as narrow test range and poor accuracy, which limit their market adoption, broader acceptance, or even clinical implementation. As such, we present potential future research directions for all types at the ending part of each subsection. Those potential directions are expected to overcome the remaining problems and move toward more ideal wearable SLMDs, which can measure sweat loss over a wide test range and hence integrate with other sweat biosensors for constructing multifunctional wearable systems. Although commercialization of wearable SLMDs is at its infancy, we believe that many commercial products will be launched in the near future based on the promising achievements in recent research.

In term of our new health perspective, the measurement of sensible and insensible sweat loss is important for two reasons. The degree of both types of sweat loss is significantly relevant for thermoregulation, skin hydration, and even emotional regulation, which indicate physiological and psychological homeostasis. However, as summarized in the previous section on advances, current research works focused mostly on measuring sweat loss due to body thermoregulation in physical activities, whereas the latter two roles have not yet received sufficient research attention by researchers. In the future, measuring sweat loss due to skin hydration and emotional regulation will provide opportunities for novel attractive applications, such as assessing cosmetic efficacy and emotional alteration.

Furthermore, it is also necessary to know the sweat rate at the time of measuring sweat components because their final measured concentrations vary with SSR in terms of component generation and sensing. Sweat biosensors capable of simultaneous measurement of SSR will be used to measure sweat component concentrations more accurately, and to determine the correlations between SSR and concentrations. These correlations not only help researchers to better understand component secretion mechanisms, but also to reduce the inter‐ and intraindividual variability by normalizing the concentration with SSR, and hence provide guidance on how to interpret concentration data reliably, which is essential for defining sweat biomarkers for diseases. Interestingly, in biomarker epidermal sensors based on interstitial fluid (ISF), some researchers have observed that calibrating the ISF biomarker concentration readouts with SSR would address potential ISF dilution by passive sweating, thereby improving the accuracy of measurement.^[^ ^201^, ^202^ ^]^ Therefore, to obtain more accurate results, researchers are encouraged to use SLMDs to determine SSR and calibrate the concentration readouts when monitoring sweat or ISF biomarkers.

In terms of long‐term development perspective of sweat sensing, there are still key challenges that should be further investigated to make sweat one of the reliable biofluids for monitoring health conditions. The main challenges are as follows:

1. Because of regional variability in sweat rate and component concentrations, it seems that data from one region cannot be applied to the entire body,^[^ ^42^ ^]^ which will increase the difficulty of measuring the sweat secretion states of the entire body using wearable devices. Nevertheless, we found that there is a degree of correlation between the forearm sweat rate and whole‐body sweat rate.^[^ ^192^, ^203^ ^]^ Thus, combining the sweat sensing data of the forearm with data from other potential regions and relevant calibration factors is expected to yield the sweat secretion state of the entire body.
2. An additional challenge is to formulate an accurate and elaborate sweat gland model to describe sweat gland and simulate sweat generation mechanism.^[^ ^53^, ^112^ ^]^ In terms of biomarker sensing, such a model will improve understanding of each biomarker partitioning and transport, as well as their correlation with blood or ISF levels; this will explore the use of sweat biomarkers for more opportunities in disease diagnostics.
3. The inter‐ and intraindividual variability in a region or entire body is due to differences in the sweat secretion rate per gland instead of the total number of active sweat glands.^[^ ^42^, ^59^ ^]^ As such, measuring sweat loss from a single gland will offer more insights into inter‐ and intraindividual variability and related diseases, such as atopic dermatitis. Moreover, combining the above sweat gland model will evaluate the correlation between sweat rates and component concentrations. Therefore, future research efforts are needed to develop a smaller SLMD capable of measuring the sweat loss of a single gland. We believe that the absorbent‐material‐based SLMD has the highest potential to achieve this.
4. The final sweat component concentrations are also affected by surface contamination regardless of high or low sweat rate.^[^ ^109^, ^204^, ^205^ ^]^ Further contamination cannot be calibrated like the SSR and needs to be reduced as much as possible by carefully cleaning the skin surface, coating the oil film, and other methods.
5. Thermal stimulation (exercise activity and passive heating) is still the main sweat sampling method in recently reported works, which makes the developed sensors unfeasible for wider use. Extensive efforts are required to exploit other innovative and reliable sweat sampling methods, and parallel efforts to investigate the effects of such methods on sweat composition are also needed. Fingertip touch sampling through hydrogel for wider applications will be the focus of sampling in future sweat sensing.^[^ ^67^, ^206^, ^207^, ^208^, ^209^ ^]^

## Conflict of Interest

The authors declare no conflict of interest.
